# TLR4-mediated IL-12 production enhances IFN-γ and IL-1β production, which inhibits TGF-β production and promotes antibody-induced joint inflammation

**DOI:** 10.1186/ar4048

**Published:** 2012-10-04

**Authors:** Hye Sung Kim, Doo Hyun Chung

**Affiliations:** 1 Department of Pathology, Seoul National University College of Medicine, 28 Yongon-dong, Chongno-gu, Seoul, 110-799, Korea; 2 Laboratory of Immune Regulation in Department of Biomedical Sciences, Seoul National University College of Medicine, 28 Yongon-dong, Chongno-gu, Seoul, 110-799, Korea

## Abstract

**Introduction:**

Toll-like receptor (TLR)4 promotes joint inflammation in mice. Despite that several studies report a functional link between TLR4 and interleukin-(IL-)1β in arthritis, TLR4-mediated regulation of the complicated cytokine network in arthritis is poorly understood. To address this, we investigated the mechanisms by which TLR4 regulates the cytokine network in antibody-induced arthritis.

**Methods:**

To induce arthritis, we injected mice with K/BxN serum. TLR4-mediated pathogenesis in antibody-induced arthritis was explored by measuring joint inflammation, cytokine levels and histological alteration.

**Results:**

Compared to wild type (WT) mice, TLR4^-/- ^mice showed attenuated arthritis and low interferon (IFN)-γ, IL-12p35 and IL-1β transcript levels in the joints, but high transforming growth factor (TGF)-β expression. Injection of lipopolysaccharide (LPS) enhanced arthritis and exaggerated joint cytokine alterations in WT, but not TLR4^-/- ^or IL-12p35^-/- ^mice. Moreover, STAT4 phosphorylation in joint cells and intracellular IL-12p35 expression in macrophages, mast cells and Gr-1^+ ^cells were detected in WT mice with arthritis and enhanced by LPS injection. Therefore, IL-12p35 appears to act downstream of TLR4 in antibody-induced arthritis. TLR4-mediated IL-12 production enhanced IFN-γ and IL-1β production via T-bet and pro-IL-1β production. Recombinant IL-12, IFN-γ and IL-1β administration restored arthritis, but reduced joint TGF-β levels in TLR4^-/- ^mice. Moreover, a TGF-β blockade restored arthritis in TLR4^-/- ^mice. Adoptive transfer of TLR4-deficient macrophages and mast cells minimally altered joint inflammation and cytokine levels in macrophage- and mast cell-depleted WT mice, respectively, whereas transfer of WT macrophages or mast cells restored joint inflammation and cytokine expression. Gr-1^+ ^cell-depleted splenocytes partially restored arthritis in TLR4^-/- ^mice.

**Conclusion:**

TLR4-mediated IL-12 production by joint macrophages, mast cells and Gr-1^+ ^cells enhances IFN-γ and IL-1β production, which suppresses TGF-β production, thereby promoting antibody-induced arthritis.

## Introduction

Rheumatoid arthritis (RA) is a chronic autoimmune disease that is characterized by persistent joint inflammation and destruction of cartilage and bone [1]. Despite intensive investigation, the immune mechanisms of RA remain unclear. Various types of immune cells, such as lymphocytes, macrophages and neutrophils, are involved in the development of joint inflammation [2]. Furthermore, a complex cytokine network is crucially implicated in the pathogenesis of RA [2]. However, the mechanism by which this complicated cytokines network is regulated in RA is not understood.

Toll-like receptors (TLRs) play crucial roles in the innate and adaptive immune systems by recognizing pathogen associated molecular patterns (PAMP) and damage associated molecular patterns (DAMP) [3]. TLR4, a prototype TLR, is complexed with MD-2 and CD14, and binds to lipopolysaccharide (LPS) [4]. Upon ligand engagement, TLR4-mediated signals are induced via toll-interleukin-1 receptor domain-containing adaptor inducing IFN-γ (TRIF) and myeloid differentiation factor 88 (MyD88) [5,6]. Several studies have demonstrated the crucial role of TLR4 in the pathogenesis of RA in murine arthritis models. TLR4 attenuated joint inflammation in IL-1 receptor antagonist-knockout (IL-1rn^-/-^) and collagen-induced arthritis (CIA) mouse models, depending on MyD88 [7]. In a zymosan-induced arthritis model, intra-articular injection of an endogenous TLR4 ligand (tenascin C) promoted joint inflammation [8]. In patients with RA, TLR4 expression is increased in synovial tissues at both early and late stages compared to those with osteoarthritis [9]. These findings suggest that TLR4-mediated signals promote joint inflammation in murine models and RA patients. With respect to the TLR4-mediated pathogenesis of RA, TLR4 inhibition reduces the severity of CIA and joint IL-1 expression [10], while IL-1-induced joint inflammation depends on TLR4 activation [11], suggesting that IL-1 signaling is associated with TLR4-mediated immune regulation in the joints. However, the mechanism by which TLR4 regulates autoimmune joint inflammation via IL-1β signals is unknown.

Among the various murine arthritis models, the K/BxN serum transfer model is a suitable *in vivo *system for exploration of the complex cellular and cytokine network in the effector phase of antibody-induced arthritis [12]. Although several reports suggest the functional link between TLR4 and IL-1β in the pathogenesis of RA, Choe *et al. *suggest that TLR4-mediated signals play a critical role in joint inflammation in the K/BxN serum transfer model, but do not depend on IL-□ production in joint tissues [13]. Therefore, the mechanism by which TLR4-mediated signals promote antibody-induced arthritis by regulating the complicated cytokine network (which includes IL-1β) in the joints remains unclear. To address this issue, we explored how TLR4 mediated signals regulate the cytokine network in the joints during antibody-induced arthritis. Here, in contrast to previous reports, we demonstrate that TLR4-mediated signals regulate joint IL-1β and IFN-γ production via IL-12 production by macrophages, mast cells and Gr-1^+ ^cells, which suppresses TGF-β production. This TLR4-mediated regulation of the cytokine network promotes antibody-induced arthritis.

## Materials and methods

### Mice

C57BL/6 mice were purchased from the Orient Company (Seoul, Korea). KRN TCR transgenic mice and NOD mice, kind gifts from Drs. D. Mathis and C. Benoist (Harvard Medical School, Boston, MA, USA) and the Institut de Genetique et de Biologie Moleculaire et Cellulaire (Strasbourg, France), were maintained on a B6 background (K/B). Arthritic mice (K/BxN) were obtained by crossing K/B and NOD (N) mice. TLR4^-/- ^mice were a generous gift from Dr. S. Akira (Osaka University, Osaka, Japan). IL-12p35^-/- ^and IL-12Rβ_2_^-/- ^mice were purchased from The Jackson Laboratory (Bar Harbor, ME, USA). These mice were bred and maintained under specific pathogen-free conditions at the Clinical Research Institute, Seoul National University (CRISNUH). Animal experiments were approved by the Institutional Animal Care and Use Committee at the CRISNUH.

### Serum transfer, arthritis scoring, and histological examination

Arthritic K/BxN mice were bled and sera were pooled. Recipient mice were injected i.p. (intraperitoneally) with 150 μL of pooled K/BxN sera on Days 0 and 2 (except for those used in two experiments to investigate the effects of LPS on joint inflammation (100 μL)). Three to six mice were used in each experimental group. Moreover, the individual mouse number in each experimental group was described in each figure legend in detail.

Ankle thickness was measured with calipers (Manostat, Herisau, Switzerland). Joint swellings in individual limbs were scored as follows: 0, no joint swelling; 1, swelling of one finger joint; 2, mild swelling of a wrist or ankle; and 3, severe swelling of a wrist or ankle. Joint swelling scores in four limbs were added up, which were expressed as clinical indexes. To examine histological changes in joint tissues, whole knee joints and hind paws were fixed in 10% formalin 10 days after K/BxN serum transfer, decalcified and embedded in paraffin. Sections were stained with H&E. Histological alterations were estimated according to criteria described previously [14]. Scores of 0 to 4 were given for joint inflammation based on the following criteria: 0, no inflammation; 1, slightly thickening of the lining layer or some infiltrating cells in the sublining layer; 2, slightly thickening of the lining layer plus some infiltrating cells in the sublining layer; 3, thickening of the lining layer influx of cells in the sublining layer; and 4, synovium highly infiltrated with many infiltration of inflammatory cells in the intra- and peri-articular areas. In addition, cartilage erosion was estimated on a scale of 0 to 4: 0, no destruction; 1, minimal erosion in single spots; 2, mild to moderate erosion in a limited area; 3, extensive erosion; and 4, general destruction. The evaluators were blinded for the experimental groups.

### Preparation for total joint cells

To prepare total joint cells, whole joint and hind paws were obtained from mice 10 days after K/BxN serum transfer. After the skin was removed, the joints were twisted with forceps. Tissues between twisted joints were taken, and then articular surfaces of the joints were scraped with sharp forceps in order to take the remaining joint cells. These joint tissues were harvested in PBS, filtered in 40 μm cell strainer (SPL Life Sciences, Pocheon, Korea), and then collected. Total joint cells contained immune cells (70%) and non-immune cells (30%) (see Additional file 1A). Furthermore, immune cells consisted of various cell subsets (see Additional file 1B). For subset analysis, PE-conjugated anti-CD45.2 (isotype control; mouse IgG_1_,κ), PE-conjugated anti-c-kit (isotype control; mouse IgG_2b_, κ), PE-Cy5-conjugated anti-mouse F4/80 (isotype control; mouse IgG_2a_, κ), FITC-conjugated anti-mouse Gr-1 (isotype control: mouse IgG_2b_, κ), PE-conjugated anti-mouse NK1.1 (isotype control; mouse IgG_1_), and PE-Cy5-conjugated anti-mouse TCRβ mAbs (isotype control; mouse IgG) were used. These antibodies were purchased from BD Pharmingen (San Diego, CA, USA) except for anti-c-kit and anti-F4/80 mAbs (e-Bioscience, San Diego, CA, USA).

### Injection of LPS and recombinant cytokines

WT B6, TLR4^-/- ^or IL-12p35^-/- ^mice were injected i.p. with 5 μg of LPS (Sigma Chemical Co., St. Louis, MO, USA) one day before K/BxN serum transfer. Recombinant (r) mouse (m) IL-12, IFN-γ and IL-1β were purchased from R&D Systems (Minneapolis, MN, USA). Injection doses of IL-12 and IFN-γ were decided based on previous report [15]. TLR4^-/- ^mice were injected i.p. with 500 ng of rmIL-12 or rmIL-1β dissolved in 300 μl of PBS one day before and after K/BxN serum transfer (Days -1, +1). TLR4^-/- ^mice were then injected i.p. with rmIFN-γ (400 ng in 300 μL of PBS) one day before K/BxN serum transfer.

### Blockade of TGF-β *in vivo *using mAb

To block TGF-β *in vivo*, WT B6 mice were injected i.p. with 100 μg of anti-TGF-β (R&D Systems) or control rat IgG mAbs (R&D Systems) one day before and one, three and five days after K/BxN serum transfer.

### Real-time PCR analysis

cDNA, prepared as described previously [16], was amplified in reactions containing TaqMan Universal Master Mix (Perkin-Elmer Biosystems, Wellesley, MA, USA), a gene-specific TaqMan probe, forward and reverse primers, and water. Gene-specific PCR products were measured using an Applied Biosystems 7500 Sequence Detection System (Perkin-Elmer Biosystems). The expressions of individual cytokines were quantified by a standard curve method and normalized to GAPDH expression. The following primers and probes were synthesized by Applied Biosystems (TagMan Predeveloped Assay Reagent): GAPDH, 4352339E; IFN-γ (Mm 00801778 m1; TGF-β_1_, GCAACATGTGGAACTCTACCAGAA (forward), GACGTCAAAAGACAGCC ACTCA (reverse), and FAM-ACCTTGGTAACCGGCTGCTGACCC-TAMRA; IL-12p35, Mm 00434169 m1; IL-1β: Mm 00434228 m1; IL-6: Mm 00446190 m1; TLR4 Mm 00445273 m1; HSP60: Mm 01289491 m1; HMGB-1: Mm 00849805 m1; fibronectin-1: Mm 01256744 m1.

### Intracellular staining for IL-12 and T-bet

Joint cells obtained from mice with antibody-induced arthritis, some of which had been injected with LPS, were filtered with 40-μm MILLEX GV filters (SPL Life Sciences, Seoul, Korea). In addition, spleen cells from TLR4^-/- ^mice were cultured with LPS (100 ng/ml) and/or recombinant IL-12 (50 ng/ml) for 4 h. After washing, these cells (1 × 10^6^) were stained with PE-conjugated anti-mouse c-kit (BD Pharmingen) or PE-cy5-conjugated anti-mouse F4/80 (eBioscience) mAb (0.5 μg in 200 μL of PBS) in the presence of anti-mouse 2.4G2 mAb (BD Pharmingen) for 30 minutes at 4°C. Anti-2.4G2 mAb is used to block immunoglobulin binding to FcγIII and FcγII on the cells. To perform intracellular staining, the cells were fixed and permeabilized with Cytofix/Cytoperm according to the manufacturer's instructions (BD Biosciences). Then, cells were stained with Alexa Fluor 647-conjugated anti-mouse IL-12p35 (BD Pharmingen) or APC-cy7-conjugated anti-mouse T-bet (eBioscience) mAb. Cells were analyzed using a FACS LSR II flow cytometer (BD Biosciences) and FlowJo software (Treestar, Inc., San Carlos, CA, USA)

### Enzyme-linked immunosorbant assay (ELISA)

Cytokine amounts were estimated in joint cell culture supernatant. To measure the IL-12 levels, joint cells were cultured with control peptide, MyD88 or TRIF inhibitor (20 μM/ml, Invitrogen, Carlsbad, CA, USA) in the presence of LPS for 24 h. ELISA kits for all cytokines were obtained from BD Biosciences and used according to the manufacturer's instructions. Standard curves were generated using purified rmIFN-γ, IL-1β and IL-12. The reaction was stopped with 3N hydrochloric acid, and the absorbance was measured at 450 and 570 nm.

### Adoptive transfer experiments

To deplete Gr-1^+ ^cells *in vivo*, 100 μg of anti-mouse Gr-1 mAb (BD Bioscience) was injected intravenously into WT mice one and three days before sacrifice. To deplete macrophages, 200 μL of liposomal vehicle (LV) and clodronate liposomes were injected into a tail vein three days before sacrifice. Clodronate liposomes were a gift from Dr. N. van Rooijen (Vrije Univiersiteit, Amsterdam, The Netherlands). WT mice were injected i.p. with compound 48/80 (Sigma Chemical Co., St. Louis, MO, USA) twice per day at the following doses to deplete mast cells: 0.5 mg/kg Day 1, 1 mg/kg Day 2, 2 mg/kg Day 3, 3 mg/kg Day 4, and 4 mg/kg Day 5. Spleen cells (1 × 10^7^) obtained from WT B6 or Gr-1^+ ^cell-depleted mice were adoptively transferred into TLR4^-/- ^mice by intravenous injection one day before K/BxN serum transfer.

### Western blot analysis

Ten days after K/BxN serum transfer, total joint cells were obtained from whole joint tissues and stimulated with LPS or rmIL-12 for 24 h. Proteins were eluted from these cells using extraction reagent (GenDepot, Barker, TX, USA), and Western blot analysis was performed as described previously [17]. The blots were subsequently incubated with rabbit anti-mouse pro-IL-1β, mouse anti-mouse STAT4, anti-pSTAT4 or anti-β-actin mAb (Cell Signaling Technology, Beverly, MA, USA). Proteins were visualized using an LAS-4000 Mini imaging system (Fujifilm Co., Tokyo, Japan).

### Statistical analysis

Statistical significance was analyzed using Prism 5.0 (GraphPad Software Inc., San Diego, CA, USA). A *t*-test was used to compare pairs of groups and one-way ANOVA followed by a Tukey's test was used. For all analyses, a *P*-value of < 0.05 was considered significant.

## Results

### TLR4-mediated signaling promotes antibody-induced arthritis

**Figure 1:**
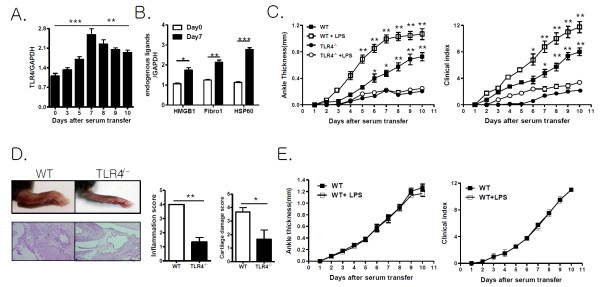
**TLR4 promotes antibody-induced arthritis**. (**A**) Toll-like receptor (TLR)4 transcript levels were measured in the ankle joint tissues of wild type (WT) mice 0, 3, 5, 7, 8, 9 and 10 days after K/BxN serum injection (100 μL, twice) by real-time PCR (*n *= 5). (**B**) Levels of transcription of endogenous TLR4 ligands were estimated in the ankle joint tissues of WT mice zero and seven days after K/BxN serum transfer (100 μL, twice) by real-time PCR (*n *= 4). (**C**) Ankle thickness and clinical scores were measured in WT and TLR4^-/- ^mice, some of which had been injected with LPS, after K/BxN serum transfer (100 μL, twice) (*n *= 6). (**D**) Gross and histological examination of ankle joints in WT and TLR4^-/- ^mice seven days after serum transfer (*n *= 3), which was scored. (**E**) Ankle thickness and clinical scores were measured in WT mice, some of which had been injected with LPS, after K/BxN serum transfer (150 μL of serum, twice) (*n *= 5). The results shown are representative of three repeated independent experiments. n.s., not significant; * *P *< 0.05, ** *P *< 0.01, *** *P *< 0.001.

To correlate joint TLR4 expression and antibody-induced arthritis, the expression of TLR4 and its endogenous ligands were analyzed in the joints of WT mice with antibody-induced arthritis by real-time PCR. TLR4 was constitutively expressed in the joints. Its expression gradually increased, peaked at Day 7, and thereafter gradually decreased (Figure 1A). Consistent with the TLR4 expression pattern in the joints, expression of endogenous TLR4 ligands, such as HSP60, HMGB1 and fibronectin, were also increased in the joints of WT mice at Day 7 after K/BxN serum transfer (Figure 1B). These findings suggest that TLR4 expression in the joints may be involved in the pathogenesis of antibody-induced arthritis. Therefore, to investigate whether TLR4 signaling affects the development of antibody-induced arthritis, we assessed joint inflammation in WT and TLR4^-/- ^mice after K/BxN serum transfer. WT mice showed measurable joint swelling four to five days after K/BxN serum transfer. This swelling peaked at 9 to 10 days after serum transfer. In contrast, TLR4^-/- ^mice were resistant to the development of joint inflammation until Day 6 and showed mild ankle swelling 6 to 10 days after K/BxN serum transfer (Figure 1C). Maximum joint swelling was much lower in TLR4^-/- ^mice than WT mice (Figure 1C, D). Histological examination of the ankle joints of WT mice at Day 7 revealed significant infiltration of inflammatory cells in the joints, whereas TLR4^-/- ^mice showed mild inflammatory cell infiltration in the ankle joints (Figure 1D). To investigate LPS-mediated TLR4 signaling in antibody-induced arthritis, we injected WT mice with an amount of K/BxN serum that resulted in sub-maximal joint swelling because LPS injection did not alter full-blown arthritis in WT mice (Figure 1C, E). Injection of LPS into WT mice exacerbated joint swelling during antibody-induced arthritis, but it did not alter joint inflammation in TLR4^-/- ^mice (Figure 1C). These results indicate that the regulation of arthritis by LPS-mediated signals is dependent on the degree of joint inflammation and TLR4 signaling may play a crucial role in the development and progression of antibody-induced arthritis.

### TLR4-mediated IL-12 production promotes antibody-induced arthritis

**Figure 2:**
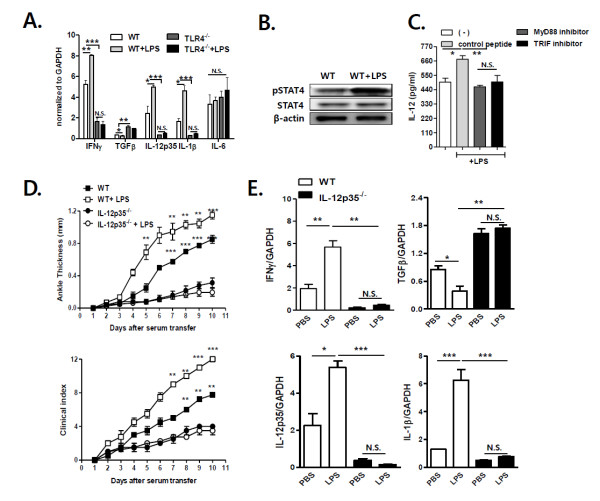
**TLR4-mediated IL-12 production by joint cells promotes antibody-induced arthritis**. To induce antibody-induced arthritis, mice were injected with K/BxN serum (100 μL) twice (**A, B, D, E**). (A) interferon (IFN)-γ, transforming growth factor (TGF)-β, interleukin(IL)-12p35, IL-1β, and IL-6 transcript levels were measured in the joints of wild type and TLR4^-/- ^mice, which had been injected with lipopolysaccharide (LPS), 10 days after serum injection (100 μL of serum, twice) by real-time PCR (*n *= 4). LPS (5 μg/300 μL in phosphate-buffered saline (PBS)) was injected intraperotineally (i.p.) one day prior to K/BxN serum transfer. (B) STAT4 and phospho-STAT4 expression in joint cells from WT mice, some of which had been injected with LPS, as analyzed by Western blotting. (**C**) Joint cells from WT mice with arthritis were incubated with LPS and a control peptide, MyD88 or TRIF inhibitor, and IL-12 levels were measured in culture fractions. (D) Ankle thickness and clinical scores were measured in WT and IL-12p35^-/- ^mice, some of which had been injected with LPS (5 μg in 300 μL of PBS) in the K/BxN serum transfer model (*n *= 6). (E) IL-4, IFN-γ, TGF-β, IL-12p35, IL-1β and IL-6 transcript levels were measured in the joints of WT and IL-12p35^-/- ^mice, some of which had been injected with LPS (5 μg in 300 μL of PBS) 10 days after K/BxN serum transfer by real-time PCR (*n *= 6). The results shown are representative of three repeated independent experiments. n.s., not significant; * *P *< 0.05, ** *P *< 0.01, *** *P *< 0.001.

To explore the mechanism by which TLR4 signals promote antibody-induced arthritis, we measured mRNA expression of various cytokines in the joint tissues of TLR4^-/- ^and WT mice, some of which had been injected with LPS, 10 days after K/BxN serum transfer. Joint TGF-β transcript levels were higher in TLR4^-/- ^mice than WT mice, whereas TLR4^-/- ^mice showed lower joint IFN-γ, IL-12p35 and IL-1β transcript levels than WT mice. In WT mice, LPS injection increased IFN-γ, IL-12p35 and IL-1β transcript levels in the joints, but reduced TGF-β □ transcript levels. In contrast, TLR4^-/- ^mice did not show altered cytokine expression in the joints as a result of LPS injection during antibody-induced arthritis (Figure 2A). IL-6 levels in joint tissues were similar in the two groups of mice during antibody-induced arthritis. These findings suggest that TLR4 promotes antibody-induced arthritis by regulating pro-inflammatory and anti-inflammatory cytokine production in the joints.

Western blotting experiments revealed that joint cells obtained from WT mice injected with LPS showed increased phosphorylation of STAT4, a transcription factor crucial for IL-12 function, as compared with cells obtained from WT mice (Figure 2B). These findings suggest that TLR4-mediated signals enhance IL-12 production in the joints during antibody-induced arthritis. Furthermore, MyD88 and TRIF inhibitors inhibited LPS-induced production of IL-12p35 in joint cells from WT mice with arthritis as compared with cells treated with a control peptide, indicating that LPS-mediated IL-12p35 production during antibody-induced arthritis depends on MyD88 and TRIF (Figure 2C). Moreover, a previous study demonstrated that IL-12p35 promotes antibody-induced arthritis by respectively enhancing and suppressing the production of IFN-γ and TGF-β in the joints [15]. Therefore, we hypothesized that IL-12p35 acts downstream of TLR4 to regulate the cytokine network in antibody-induced arthritis. To address this hypothesis, we compared WT and IL-12p35^-/- ^mice in terms of joint swelling and cytokine production in the presence or absence of LPS during antibody-induced arthritis. In contrast to WT mice, administration of LPS to IL-12p35^-/- ^mice altered neither joint swelling nor IL-1β, IFN-γ or TGF-β transcript levels in the joints (Figure 2D, E). Collectively, these data indicate that LPS-induced TLR4 signals promote antibody-induced arthritis by inducing the production of IL-12p35 in the joints, which may regulate the complex cytokine network in the joints.

### TLR4-mediated IL-12 production enhances IL-1β and IFN-γ production in the joints, which suppresses TGF-β production, and thereby promotes antibody-induced arthritis

**Figure 3:**
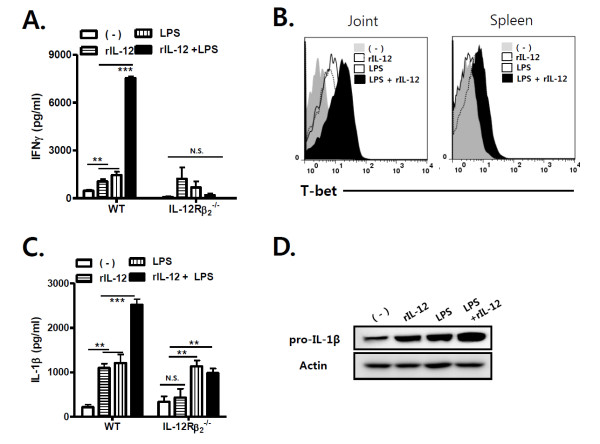
**TLR4-mediated IL-12 production enhances joint IFN-γ and IL-1β production in antibody-induced arthritis**. (**A **and **C**) Spleen cells from WT or interleukin(IL)-12Rβ_2_^-/- ^mice were stimulated with LPS and/or recombinant IL-12, and amounts of IFN-γ and IL-1β were measured in the culture fraction by ELISA. (**B**) Intracellular T-bet expression was measured in joint cells from WT mice, some of which had been stimulated with LPS and/or recombinant IL-12, by flow cytometry. In addition, spleen cells from TLR4^-/- ^mice were stimulated with LPS and/or recombinant IL-12. (**D**) Joint cells from WT mice with antibody-induced arthritis were stimulated with LPS and/or recombinant IL-12. Western blotting was performed to measure amounts of pro-IL-1β in these joint cells as compared with β-actin levels. The results shown are representative of three repeated independent experiments. n.s. not significant; * *P *< 0.05, ** *P *< 0.01, *** *P *< 0.001.

Next, to investigate whether TLR4-mediated IL-12p35 production regulates IFN-γ and IL-1β production in the joints during antibody-induced arthritis, spleen cells were obtained from WT and IL-12Rβ_2_^-/- ^mice, and cultured with LPS and/or recombinant IL-12 *in vitro *(Figure 3A, C). Both LPS and recombinant IL-12 increased the production of IFN-γ and IL-1β by WT spleen cells. LPS-mediated IL-1β and IFN-γ production by spleen cells was further enhanced by recombinant IL-12. In IL-12Rβ2-deficient spleen cells, recombinant IL-12 did not alter the production of both IL-1β and IFN-γ, while LPS alone increased IL-1β production. Consistent with these results, injection of LPS or recombinant IL-12 increased T-bet expression in joint cells from WT mice with arthritis compared with those from non-LPS-treated WT mice. Co-stimulation of LPS and recombinant IL-12 produced an even greater response (Figure 3B). Furthermore, recombinant IL-12 increased T-bet expression in spleen cells from TLR4^-/- ^mice in the presence or absence of LPS, whereas LPS did not affect T-bet expression. Pro-IL-1β is induced by TLR signaling (signal 1), cleaved into IL-1β by caspase-1 activity (signal 2) in the cytoplasm of immune cells, and secreted as an active protein [18]. Western blotting revealed that recombinant IL-12 increased pro-IL-1β expression in joint cells from WT mice with arthritis in the presence or absence of LPS, suggesting that TLR4-mediated IL-12 regulates the production of pro-IL-1β in joint cells, rather than its cleavage (Figure 3D). These results suggest that TLR4-mediated IL-12 production increases the production of both IFN-γ and IL-1γ in the joints during antibody-induced arthritis.

**Figure 4:**
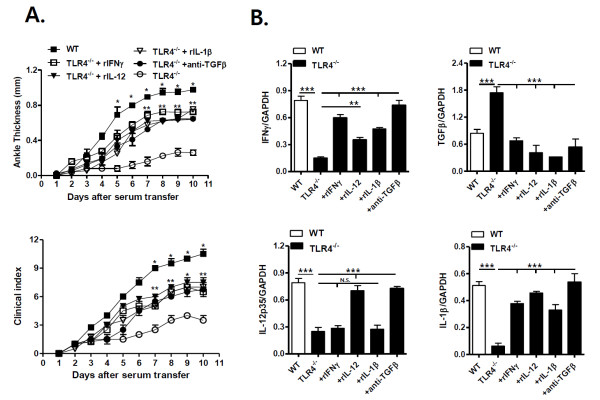
**Joint IFN-γ and IL-1β production via TLR4-mediated IL-12 production suppresses TGF-β production, thereby promoting antibody-induced arthritis**. (**A**) Toll-like receptor (TLR)4^-/- ^mice were injected intraperitoneally (i.p.) with recombinant interleukin (IL)-12, IL-1β (500 ng in 300 μL of PBS), interferon (IFN)-γ (400 ng in 300 μL of phosphate-buffered saline (PBS)) or anti-transforming growth factor (TGF)-β1 mAb (100 μg in 300 μL of PBS), one and three days prior to, and one day after K/BxN serum transfer (150 μL, twice). Clinical scores and ankle thickness were monitored in these mice and compared with those in TLR4^-/- ^and wild type (WT) mice (*n *= 4). (**B**) IFN-γ, TGF-β, IL-12p35 and IL-1β transcript levels were measured in the joint tissues of WT and TLR4^-/- ^mice, and TLR4^-/- ^mice injected with recombinant IL-12, IL-1β, IFN-γ or anti-TGF-β1 mAb (*n *= 4). The results shown are representative of three repeated independent experiments. n.s., not significant; * *P *< 0.05, ** *P *< 0.01, *** *P *< 0.001.

To confirm the functional involvement of individual cytokines in TLR4-mediated arthritis, we injected i.p. recombinant IFN-γ, IL-12 or IL-1β into TLR4^-/- ^mice during antibody-induced arthritis. Injection of recombinant IFN-γ, IL-12 or IL-1β into TLR4^-/- ^mice restored arthritis as compared to WT mice (Figure 4A), indicating that these pro-inflammatory cytokines contribute to the pathogenesis of TLR4-mediated joint inflammation in antibody-induced arthritis. Consistent with the results of our *in vitro *experiments (Figure 3A-D), recombinant IL-12 increased the expression of IFN-γ and IL-1β in the joints of TLR4^-/- ^mice with arthritis, whereas neither recombinant IL-1β nor IFN-γ altered joint IL-12p35 expression levels (Figure 4B). These findings suggest that IL-12p35 acts upstream of IL-1β and IFN-γ in the joints during antibody-induced arthritis. Meanwhile, the administration of recombinant IL-1β, IL-12 or IFN-γ to TLR4^-/- ^mice reduced TGF-β transcript levels in the joints during antibody-induced arthritis, indicating that these pro-inflammatory cytokines inhibit joint TGF-β production (Figure 4B). Furthermore, anti-TGF-β mAb-induced TGF-β blockade in TLR4^-/- ^mice increased joint swelling and IL-1β, IL-12p35 and IFN-γ mRNA levels in the joints (Figure 4A, B), indicating that TGF-β production suppresses joint inflammation in TLR4^-/- ^mice. It further appears that TLR4-mediated signals regulate joint inflammation by altering the balance between TGF-β and pro-inflammatory cytokine production in the joints. Taken together, these findings suggest that TLR4-mediated IL-12 production enhances joint production of IL-1β and IFN-γ, which suppresses TGF-β production and, thereby, promotes antibody-induced arthritis.

### TLR4-mediated IL-12 production by macrophages and mast cells plays a crucial role in promoting antibody-induced arthritis, whereas Gr-1^+ ^cells partially contribute to TLR4-mediated joint inflammation

**Figure 5:**
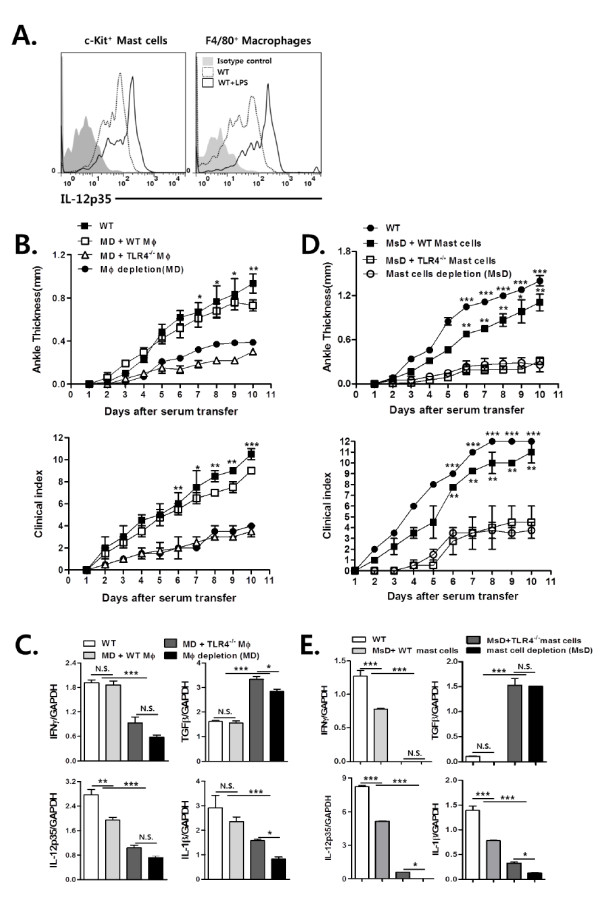
**TLR4-mediated IL-12 production by joint macrophages and mast cells plays crucial roles in promoting antibody-induced arthritis**. To induce antibody-induced arthritis, mice were injected with K/BxN serum (150 μL) twice (**A-E**). (A) Intracellular interleukin(IL)-12 expression was estimated in c-kit^+ ^mast cells and F4/80^+ ^macrophages from wild type (WT) mice, some of which had been injected with lipopolysaccharide (LPS) 10 days after K/BxN serum transfer. (B and C) Macrophages from WT or Toll-like receptor (TLR)4^-/- ^mice were adoptively transferred into macrophage-depleted WT mice with antibody-induced arthritis. (D and E) Mast cells from WT or TLR4^-/- ^mice were adoptively transferred into mast cell-depleted WT mice with antibody-induced arthritis. (B and D) Ankle thickness and clinical scores were measured. (C and E) Interferon (IFN)-γ, transforming growth factor (TGF)-β, IL)-12p35, and IL-1β transcript levels in the joints 10 days after K/BxN serum transfer were measured by real-time PCR. The results shown are representative of three repeated independent experiments. n.s., not significant; * *P *< 0.05, ** *P *< 0.01, *** *P *< 0.001; B, C, D, and E, *n *= 3.

**Figure 6:**
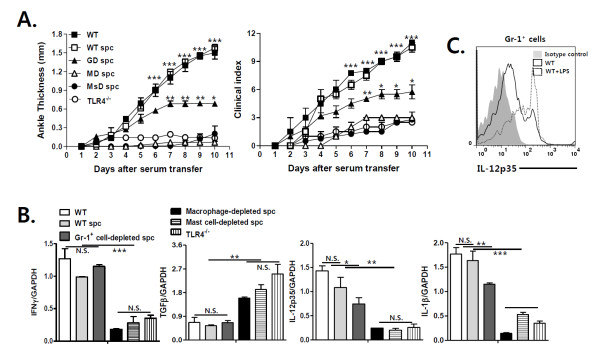
**Gr-1^+ ^cells contribute to TLR4-mediated pathogenesis in antibody-induced arthritis**. To induce antibody-induced arthritis, mice were injected with K/BxN serum (150 μL) twice (**A-C**). (A) Toll-like receptor (TLR)4^-/- ^mice were adoptively transferred with spleen cells (spc) from wild type (WT) mice some Gr-1^+ ^cell- (GD), macrophage- (MD), and mast cell- (MsD) depleted, one day prior to K/BxN serum transfer. Ankle thickness and clinical scores were monitored in each group and compared with those in wild type (WT) and TLR4^-/- ^(TLR4^-/-^) mice (*n *= 3). (B) Interferon (IFN)-γ, transforming growth factor (TGF)-β, interleukin (IL)-12p35 and IL-1β transcript levels in the joints of each group 10 days after K/BxN serum transfer (*n *= 3) were measured by real-time PCR. (C) Intracellular IL-12 expression in Gr-1^+ ^cells from WT mice, some of which had been injected with LPS, was measured 10 days after K/BxN serum transfer (*n *= 3). The results shown are representative of three repeated independent experiments. n.s., not significant; * *P *< 0.05, ** *P *< 0.01, *** *P *< 0.001.

To determine whether joint immune cells produce IL-12 via TLR4 signals during arthritis, we performed intracellular staining for IL-12p35 in joint macrophages and mast cells from WT mice with antibody-induced arthritis, some of which had been injected with LPS. Among the different joint immune cells, macrophages and mast cells that express TLR4 are crucial in the development of antibody-induced arthritis [19,20]. Intracellular staining and flow cytometric analysis revealed that IL-12p35 was produced by macrophages and mast cells from WT mice with arthritis, and that this production was enhanced by LPS injection (Figure 5A). Next, to confirm the function of macrophages and mast cells in TLR4-mediated regulation of arthritis, we transferred macrophages and mast cells from WT or TLR4^-/- ^mice into macrophage- and mast cell-depleted WT mice, respectively. In WT mice, depletion of macrophage or mast cells attenuated antibody-induced joint inflammation and reduced IFN-γ, IL-12 and IL-1β expression in the joints, but increased joint TGF-β expression. Adoptive transfer of WT macrophages or mast cells reversed these changes (Figure 5C, E). However, TLR4-deficient macrophages and mast cells did not alter joint inflammation or cytokine expression in joint tissues in these mice (Figure 5B, D). Consistent with these results, adoptive transfer of macrophage- (MD) or mast cell-depleted (MsD) WT spleen cells into TLR4^-/- ^mice did not restore antibody-induced arthritis or cytokine production in the joints, whereas non-depleted WT spleen cells fully restored arthritis in TLR4^-/- ^mice (Figure 6A, B). Gr-1^+ ^cell-depleted spleen cells partially restored joint inflammation, indicating that Gr-1^+ ^cells partly contribute to the TLR4-mediated pathogenesis of arthritis. Nevertheless, flow cytometric analysis revealed that joint Gr-1^+ ^cells in WT mice with antibody-induced arthritis expressed intracellular IL-12p35, whose levels were increased by the injection of LPS (Figure 6C). Taken together, these results suggest that TLR4-mediated IL-12 production by macrophages, mast cells and Gr-1^+ ^cells enhances joint production of IFN-γ and IL-1β, which suppresses TGF-β production, and thereby promotes antibody-induced arthritis.

## Discussion

Several studies have demonstrated that TLR4-mediated signals induce macrophages, dendritic cells and synovial cells from RA patients to produce IL-12 *in vitro *[21-23], indicating that TLR4-mediated signals induce IL-12 production by various immune and non-immune cells. Moreover, another study demonstrated that an IL-12p35/IFN-γ axis promotes antibody-induced joint inflammation by suppressing TGF-β production in joint tissues [15]. These findings led us to hypothesize that a TLR4-mediated IL-12p35/IFN-γ axis regulates antibody-induced arthritis by suppressing TGF-β production. Consistent with this hypothesis, our current experiments revealed that IFN-γ, IL-12p35 and IL-1β transcript levels in joint tissues increased in WT mice compared with TLR4^-/- ^mice following K/BxN serum transfer, whereas TGF-β transcript levels decreased. These findings suggest that IL-1β in addition to the IL-12p35/IFN-γ axis promotes TLR4-mediated joint inflammation. Several lines of evidence in our experiments suggest that IL-12 acts downstream of TLR4, triggering the production of both IFN-γ and IL-1β in joint tissues during antibody-induced arthritis, but suppressing TGF-β production. First, TLR4^-/- ^mice produce minimal amounts of IL-12p35 in their joints during antibody-induced arthritis compared with WT mice. Moreover, injection of recombinant IL-12 into TLR4^-/- ^mice restored joint inflammation. *In vitro *experiments revealed that LPS induced IL-12 production by joint immune cells, a response dependent on MyD88 and TRIF. Injection of LPS into WT mice increased the phosphorylation of the IL-12-inducing transcription factor STAT4 in joint immune cells during antibody-induced arthritis. Collectively, these findings suggest that TLR4-mediated signals induce the production of IL-12 by joint immune cells during antibody-induced arthritis. Second, injection of LPS enhanced antibody-induced arthritis and the production of IFN-γ and IL-1β in the joints of WT mice, but not IL-12p35^-/- ^mice. Furthermore, injection of recombinant IL-12 into TLR4^-/- ^mice enhanced the production of IFN-γ and IL-1β in the joints during antibody-induced arthritis, whereas recombinant IFN-γ and IL-1β did not enhance IL-12p35 production. Moreover, LPS-induced production of IL-12 by joint immune cells increased IFN-γ and IL-1β production by enhancing T-bet expression and pro-IL-1β production. These findings suggest that TLR4-mediated IL-12 production enhances the production of both IL-1β and IFN-γ in the joints during antibody-induced arthritis. However, that IL-12 induces IL-1β production by enhancing pro-IL-1β production during joint inflammation has not previously been reported. Third, injection of recombinant IFN-γ, IL-1β or IL-12 into TLR4^-/- ^mice reduced TGF-β production in joint tissues during antibody-induced arthritis, while a TGF-β blockade restored antibody-induced arthritis in TLR4^-/- ^mice. These findings indicate that TLR4-mediated IL-12/IL-1β and IL12-/IFN-γ axes in the joints suppress TGF-β production, thereby promoting antibody-induced arthritis. As no previous reports have addressed functional links between TLR4 and IL-12 regulatory axes in the pathogenesis of antibody-induced arthritis, this study provides the first demonstration that TLR4-mediated IL-12 promotes arthritis by regulating the production of both IL-1β and IFN-γ, thereby suppressing TGF-β production.

It has been suggested that TLR4-mediated signals promote joint inflammation by increasing levels of either IL-17 or IL-1β in murine arthritis models [10,11,24,25]. However, WT and IL-17^-/- ^mice showed similar joint inflammation and cytokine production in the K/BxN serum transfer model [15], suggesting that IL-17 may have minimal involvement in the TLR4-mediated regulation of antibody-induced arthritis. With regard to IL-1β, Choe *et al. *suggested that TLR4 regulation of joint inflammation bypasses the need for IL-1, although TLR4 and IL-1R play crucial roles in promoting antibody-induced arthritis [13]. In their experiments, IL-1R^-/- ^mice showed attenuated arthritis compared with WT mice upon K/BxN serum transfer, while LPS injection did not alter joint inflammation in IL-1R^-/- ^or WT mice. Based on these findings, they suggested that LPS-mediated TLR4 signals do not regulate joint inflammation in WT or IL-1R^-/- ^mice. In contrast to their results, our experiments demonstrated that injection of WT mice with LPS aggravated arthritis, when sub-maximal joint swelling was induced by injection of an appropriate amount of K/BxN serum, whereas LPS did not alter full-blown arthritis in WT mice (Figure 1E), a result consistent with the results of Choe *et al. *These findings suggest that LPS-mediated TLR4 signals regulate antibody-induced arthritis, depending on the severity of joint inflammation, which might also account for contradictory results that TLR4^-/- ^mice showed K/BxN serum-induced arthritis comparable to WT mice [26], although these divergent findings should be further investigated. Therefore, we do not completely rule out the possibility that IL-1β contributes to TLR4-mediated pathogenesis in antibody-induced arthritis. Consistent with this suggestion, Ji *et al. *demonstrated that joint IL-1β expression levels were significantly increased three to six days after K/BxN serum transfer and suggested that IL-1 and TNF-β play critical roles in antibody-induced arthritis [25]. Furthermore, our experiments demonstrated that recombinant IL-1β restored joint inflammation in TLR4^-/- ^mice, indicating that IL-1β promotes antibody-mediated joint inflammation, depending on TLR4-mediated immune responses.

Our data indicate that monocytes from HCV patients are activated in vivo. This interferes with their differentiation into DC, leading to deficient TLR4 signaling in these cells that are enable to induce a Th1 response. This specific defect is linked to the activation of the MEK/ERK pathwayTLR4 is expressed not only in joint-infiltrating immune cells, but also in non-hematopoietic joint tissues, and regulates joint inflammation by mediating the production of various cytokines [27,28]. Several studies have reported that macrophages, mast cells, NKT cells and Gr-1^+ ^cells play crucial roles in antibody-induced arthritis, and express TLR4 on the cell surface [16,17,19,20,29]. Our experiments demonstrated that adoptive transfer of WT mast cells or macrophages fully restored joint inflammation in macrophage- and mast cell-depleted WT mice, respectively, indicating that TLR4-expressing macrophages and mast cells, rather than non-hematopoietic joint cells, are crucial to antibody-induced arthritis. However, adoptive transfer of Gr-1^+ ^cell-depleted spleen cells only partially restored joint inflammation in TLR4^-/- ^mice, indicating that Gr-1^+ ^cells contribute less significantly to the promotion of antibody-induced arthritis via TLR4-mediated signals than macrophages and mast cells, although Gr-1^+ ^cells play a crucial role in the development of joint inflammation. With respect to NKT cells, our recent study clearly demonstrated that invariant NKT cells express TLR4, which promotes antibody-induced arthritis [30], although the expression patterns of TLR4 in NKT cells are controversial [31-33]. Therefore, macrophages, mast cells, Gr-1^+ ^cells and invariant NKT cells promote antibody-induced arthritis by expressing TLR4. Furthermore, levels of TLR4, which was constitutively expressed in the joints, gradually increased, peaked, and then gradually decreased in our current experiments. Consistent with the TLR4 expression pattern in the joints, levels of the endogenous TLR4 ligands HSP60, HMGB1 and fibronectin were also increased in the joint tissues of WT mice during antibody-induced arthritis. Moreover, antibody-induced arthritis was developed in WT, but not in TLR4^-/- ^mice in the absence of exogenous TLR4 ligand, indicating that TLR4 endogenous ligands contribute to developing antibody-induced arthritis. Therefore, TLR4 on immune cells may be engaged by endogenous or exogenous ligands, which induce TLR4-mediated downstream immunological events. Consistent with our results, levels of endogenous TLR4 ligands, including HMGB-1, s100 proteins and hyaluronic acid were found to be elevated in the synovial fluid or serum of RA patients [34], and concentrations were correlated with clinical scores in RA patients [35].

For therapeutic purposes, it would be beneficial to inhibit TLR4 signals, IL-12 production, and the effects of IL-12 on IL-1β and IFN-γ production in antibody-induced joint inflammation. Several studies have demonstrated that anti-IL-12 mAbs ameliorate CIA in mice [36,37], suggesting that a blockade of IL-12 with a neutralizing mAb may be a useful therapeutic strategy for rheumatoid arthritis. Alternatively, strategies to block the functional activity of TLR4-expressing effector cells may also be helpful in treating rheumatoid arthritis.

## Conclusions

Our experiments suggest that TLR4-mediated signals activated by endogenous or exogenous ligands induce the production of IL-12 by macrophages, mast cells and Gr-1^+ ^cells, which enhance IL-1β and IFN-γ production, thereby suppressing TGF-β production. This TLR4-mediated regulation of the cytokine network promotes antibody-induced arthritis. These findings may facilitate the development of new TLR4-targeted therapeutic strategies to inhibit rheumatoid arthritis.

## Abbreviations

CIA: collagen-induced arthritis; DAMP: damage associated molecular patterns; IL: interleukin; INF: interferon; i.p.: intraperotineally; K/BxN mice: mice obtained by crossing KRN transgenic mice of B6 background and NOD (N) mice; LPS: lipopolysaccharide; LV: liposomal vehicle; MD: microphage-depleted; MsD: mast cell-depleted; MyD88: myeloid differentiation factor 88; PAMP: pathogen associated molecular patterns; PBS: phosphate-buffered saline; RA: rheumatoid arthritis; TGF: transforming growth factor; TLRs: Toll-like receptors; TRIF: toll-interleukin-1 receptor domain-containing adaptor inducing IFN-γ.

## Competing interests

The authors declare that they have no competing interests.

## Authors' contributions

DHC made contributions to design, analysis and interpretation of data, and the writing of the manuscript. HSK made contributions to design and performing experiments, and acquisition and interpretation of data. All authors have read and approved the final manuscript for publication.

## Supplementary Material

Additional file 1 **Subset analysis for joint cells from mice with antibody-induced arthritis**. Total joint cells were obtained from WT mice seven days after K/BxN serum transfer and analyzed for cell subsets. (**A**) Total cells were stained using anti-CD45.2 mAb as compared with isotype-matched control. (**B**) Subset analysis for total joint cells was performed. Click here for file
