# Supplementary figures and images for: TLR4-mediated IL-12 production enhances IFN-γ and IL-1β production, which inhibits TGF-β production and promotes antibody-induced joint inflammation
Source: Arthritis Res Ther. 2012 Oct 4;14(5):R210. doi: 10.1186/ar4048 (PMC3580522; doi:10.1186/ar4048)

# Supplemental data 1

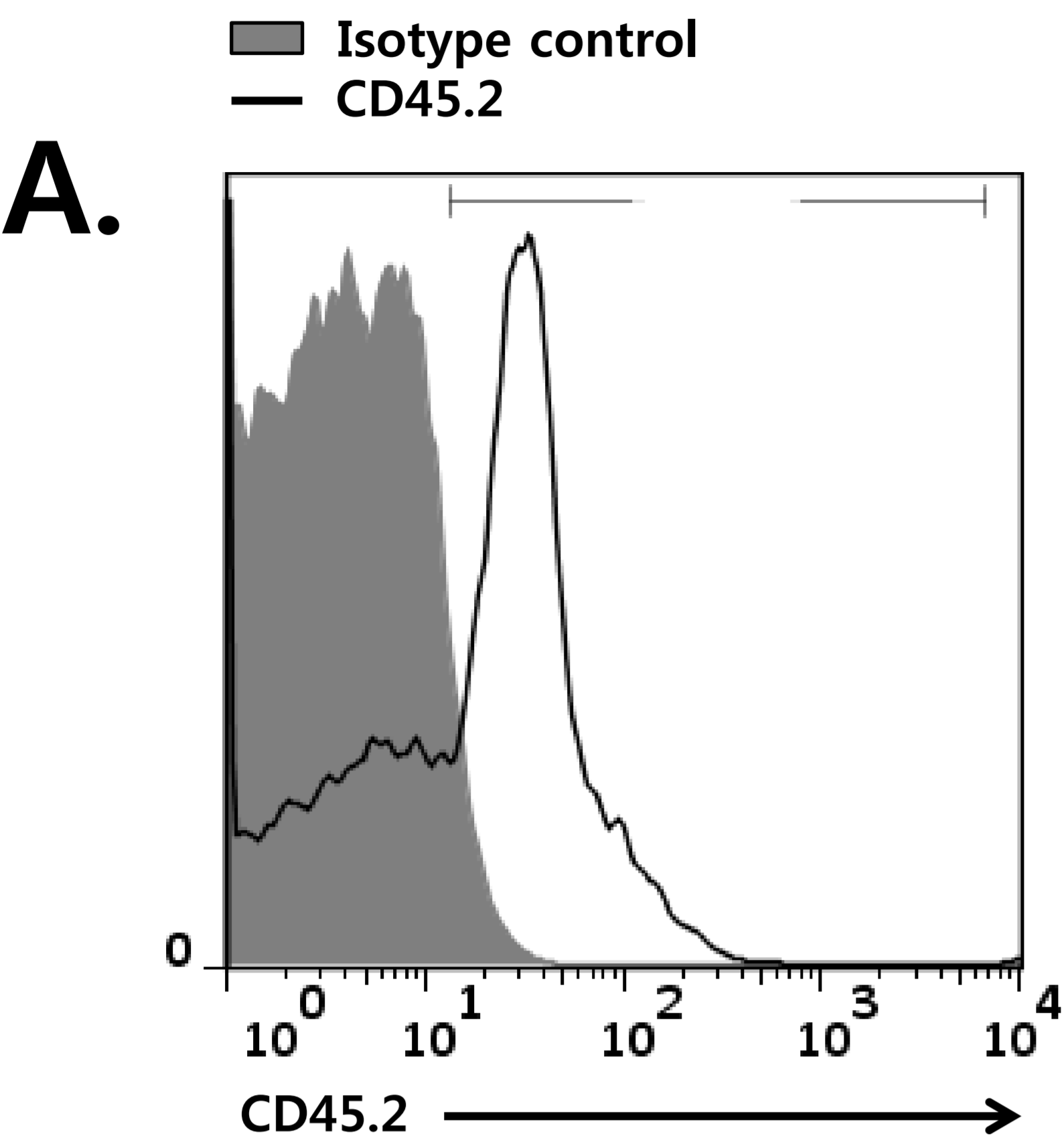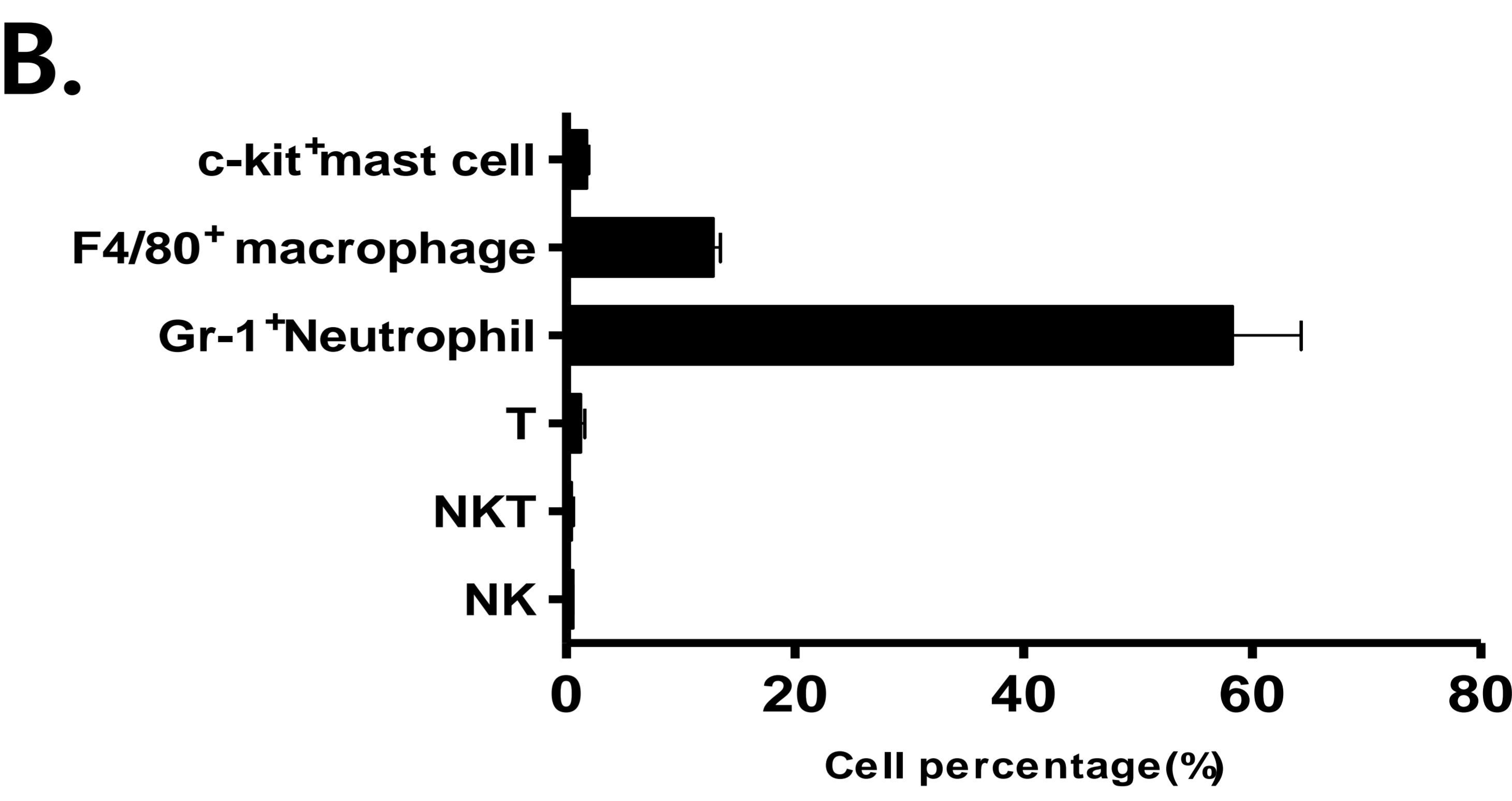

Supplement: Additional file 1 — Subset analysis for joint cells from mice with antibody-induced arthritis. Total joint cells were obtained from WT mice seven days after K/BxN serum transfer and analyzed for cell subsets. (A) Total cells were stained using anti-CD45.2 mAb as compared with isotype-matched control. (B) Subset analysis for total joint cells was performed. [file ar4048-S1.PDF]
